# Religion and Death in the United States: A Meta-Regression Comparative Assessment of Between-County Mortality Heterogeneity in the United States

**DOI:** 10.3390/ijerph19020757

**Published:** 2022-01-11

**Authors:** Frances Sissamis, Karina Villalba, Jordan Garcia, Vickie Melus, Emily J. Markentell, Ligia D. Perez, Gilbert Ramirez

**Affiliations:** 1 School of Public Health, Texas A&M University, College Station, TX 77843, USA; sissamisfrances@tamu.edu (F.S.); emilymarkentell@yahoo.com (E.J.M.); 2 Department of Population Health Sciences, University of Central Florida, Orlando, FL 32827, USA; karina.villalba@ucf.edu; 3 Health Science Center, Texas A&M University, Bryan, TX 77807, USA; jordan.garcia.6525@gmail.com; 4 Robert Stempel College of Public Health and Social Work, Florida International University, Miami, FL 33199, USA; melusvickie5@gmail.com; 5 Herbert Wertheim College of Medicine, Florida International University, Miami, FL 33199, USA; lidpere@fiu.edu

**Keywords:** religion, mortality, population health determinant, meta-regression

## Abstract

Religion can have a favorable impact on individual-level health. The influence of religion on population health, however, remains less clear. This study investigated the association between religion and mortality at the population-level. Using county data, a meta-regression was performed to examine between-county mortality heterogeneity. The percent heterogeneity associated with religion variables were compared to demographics (i.e., place, race, language, age, and gender) and health factors (i.e., individual behaviors, clinical care, social and economic, and physical environment) as predictors of mortality. Religion was measured in terms of adherence (i.e., prevalence attending/belonging to a congregation), congregation density, and the diversity of adherents and congregation by denominations. Results showed counties with lower mortality were associated with higher proportions of religion adherents and a greater diversity of adherents and congregations. Counties with higher mortality were associated with higher religion congregation density. Religion, as a parsimonious multivariate model with all demographic and health factor predictors, had less added value when controlled for individual variables or constructs. The direction of association between religion and mortality was consistent, even when controlling for demographics and health factors, and thus merits further consideration as a population health determinant, as it may play a critical role in understanding other population health outcomes.

## 1. Introduction

Religion, however defined, is both ubiquitous and variant across populations; a simple road trip is often sufficient to reveal how ubiquitous and variant religion is in the United States. A road trip would also reveal considerable demographic variation such as population densities, race, and poverty/wealth; it would only be natural to wonder if these variances are related. There is considerable evidence of an individual-level relationship between religion and health, mostly with respect to mental and physical health and from a clinical perspective. However, the relationship at a population level has not been studied as extensively as it has at the individual level. The extant individual-level evidence and the ubiquity/variance of religion across populations warrants further study of religion as a determinant of population health [1].

Based on a comprehensive review of the literature, Koening has proposed two theoretical causal pathways regarding religion/spirituality (R/S) and mental and physical health/longevity [2]. R/S may provide resources for coping with stress, via doctrines, on how to live life and how to treat others and encourage prosocial behaviors that buffer stress and leads to human support when support is needed. R/S may influence physical health/longevity through pathways such as psychological (facilitates coping and imbues negative events with meaning and purpose), social (greater social support, greater marital stability, less crime/delinquency, and greater social capital), and health behaviors (less alcohol and drug use, less cigarette smoking, more physical activity and exercise, better diet, and safer sexual practices). These two pathways (as noted by the author) are based on Western monotheistic religions (Christianity, Judaism, and Islam) and may not necessarily apply to Eastern religious traditions or the secular Humanism tradition.

Public health has been critiqued for a lack of formal theoretical models as one factor that has led to distraction from its mission of ensuring the conditions in which people can be healthy, and thus resulting in the failure of population health in the United States [3]. The critique argues that public health research tends to be focused on the *what* (empirical models) rather than the *why* or *how* (conceptual models); yet the importance of the *what* remains particularly in the early stages of research. The two “Koenig models” describe the *why* and *how* of religion and health at the individual level; population-level conceptual models are few and tend to be more general. A brief review of three population-level models follows.

The first model portrays religion and population health as a “transformative ensemble” with seven “interlocking ideas”. The seven “ideas” are labeled: (1) an embodied religious mind; (2) religious health assets; (3) leading causes of life; (4) health worlds; (5) congregate strengths; (6) boundary leadership; and (7) healthy political economy [4]. The “interlocking ideas” are centered around a concept of “deep accountability”. A key idea of this model is “religious health assets”, which the authors describe as a critical shift in thinking to “cultivate an active curiosity about the assets for health, tangible and intangible, that are already held and utilized by those seeking health for whom public health interventions are intended, and how one can leverage such aspects for health”. As a population health determinant, variance across populations in terms of prevalence of adherence to any religion (or to a specific religion), and access to congregations as places of worship or social gathering could be conceived as the “religious health assets” of a population and is thus a potential area for study.

The second model describes the relationship between religion and population health from perspectives already well-established in the public health mindset: social support, social control, and/or as social capital [5]. This model offers a broader vision of religion as a primary determinant of population health, both positive and negative, alongside economic inequality. For example, religion may reduce economic inequality by invoking charitable impulses and compassionate re-attention to the differences between individuals and between groups. Religion may also moderate the health consequences of income inequality by alleviating its psychological effect.

The third model describes religion as a “collective culture” with respect to population health [6]. It focuses first on the relationship at an individual level (in terms of beliefs, practices, feelings, and coping influencing health behaviors and disease detection/treatment, social connections and support, and mental health and character strengths) and then on three intermediary effects influencing immune, endocrine, and cardiovascular functions, which then ultimately influence physical health and longevity. The individual relationship model is then expanded to describe how community-level religion/spirituality also influences individual health as a “collective culture”. We believe that the concept of a religion collective culture may offer a more comprehensive view of public health and provide a better understanding of the connection between religion and health. This can be achieved by demonstrating the utility at the county level, such as well-defined geographic boundaries defining population membership, and the availability of county-level religion and health data (e.g., U.S. Religion Census and the University of Wisconsin Population Health Institute’s County Health Rankings and Roadmaps).

Conceptual models are necessary but not sufficient in establishing the nature of any relationship between population health and religion; empirical data, the *what*, is also required. The *what* will answer questions such as: (1) is religion ubiquitous and variant across populations; (2) how does population health vary with religion, and then how well does religion variation explain population health variance; and (3) does any relationship between religion and population health outcomes remain when controlling for more traditional population health determinants? These are complex questions for research as they lead to more questions such as how best to measure population health and religion. 

This article focuses on population health measured as mortality. Religion is measured in terms of adherence (prevalence attending/belonging to a congregation), congregation density, and the diversity of adherents and congregation by denominations. The nature of any relationship between mortality and religion will be compared to well-established relationships between mortality and demographic and health factors at the county level.

Meta-regression with county data (in lieu of study data) is used to measure mortality heterogeneity across U.S. counties and the percent heterogeneity explained by religion, demographic, and health factor variables. Religion data were obtained from the 2010 U.S. Religion Census; mortality, demographic, and health factor data were obtained from the PHI County Health Rankings & Roadmaps. Demographic constructs include measures of place, race, and gender. Health factor constructs include individual behaviors, clinical care, social and economic factors, and physical environment. Heterogeneity explained is examined as single and multivariate covariates of mortality. The research questions include:

1. What is the direction and strength of the association, if at all, between religion variables and mortality, uncontrolled and controlled for county demographics and health factors?
2. Do religion variables explain less, more, or comparable levels of population mortality heterogeneity as do demographic and health factor variables?
3. Does the inclusion of religion variables have added value when studying population mortality controlling for demographics and health factors?

## 2. Materials and Methods

### 2.1. Data 

Data obtained from the 2010 U.S. Religion Census included the number of adherents, the number of congregations, and county population size [7]. The census data were reported by state, by county, and by individual congregation. Summary statistics were also reported as an overall total (any denomination) and by six major denominations: Evangelical, Mainline Protestant, Black Protestant, Catholic, Orthodox, and Other [8]. Number of adherents were reported by individual congregations within each county and defined as including those with an affiliation to the congregation (children, members, and attendees who are not members); congregation was defined as a group of people who meet regularly (typically weekly or monthly) at a pre-announced time and location and may be churches, mosques, temples, or other meeting places [9].

Mortality, demographic, and health factor data were obtained from the County Health Rankings and Roadmaps website, which is a program of the University of Wisconsin Population Health Institute, funded by the Robert Wood Johnson Foundation [10]. County data are compiled from multiple sources over time and used to rank the health of individual counties within each state (a detailed description of individual data used for this research is provided as an Appendix; additional information is available at their website). Demographic and health factor data include:

- Demographic
  ○ Rurality;
  ○ Race and Language: African American (non-Hispanic), American Indian or Alaskan Native, Asian, Hispanic, Native Hawaiian or other Pacific Islander, White (non-Hispanic), English language proficiency;
  ○ Age and Gender: age less than 18, age 65 and older, female.
- Health Factors
  ○ Individual Behaviors: adult smoking, adult obesity, food environment index, physical inactivity, access to exercise opportunities, excessive drinking, alchohol-impaired driving deaths, sexually transmitted infections, teen births;
  ○ Clinical Care: uninsured, primary care physicians, dentists, preventable hospital stays, diabetes monitoring, mammography screening;
  ○ Social and Economic: high school graduation, some college education, unemployment, children in poverty, income inequality, inadequate social support, single parent households, violent crime, injury deaths;
  ○ Physical Environment: air pollution, drinking water violations, severe housing problems, driving alone to work, long commute when driving alone.

### 2.2. Variables for Descriptive Analyses 

The proportion of adherents for each county was estimated as the number of adherents divided by the county population size, as reported in the 2010 U.S. Census. When the proportion was greater than 1 (29 counties, particularly for congregations near a county line reporting adherents from an adjacent county), the county’s proportion of adherents was capped at 1. Congregation density was estimated as the number of congregations per 100,000 population. Diversity of religion was estimated both for adherents and for congregations using formulas reported elsewhere as Religious Diversity Index and Denominational Pluralism, respectively [11,12]. Both formulas are variants based on the Herfindahl–Hirschman Index, and values range from 0 to 1, with 0 reflecting no diversity and 1 an equal distribution of adherents and denominations across the six denomination major groups.

Mortality was estimated for each county as the average numerator (number of deaths over two time periods) divided by the average denominator (population for two time periods) per 100,000 population. All demographic and health factor variables were analyzed descriptively using their reported metrics (averaging when reported over multiple years—see Appendix).

### 2.3. Variables for Meta-Regression Analyses

The mortality numerators and denominators were used to estimate double arcsin effect sizes and standard errors [13,14]. County population size and congregation density were converted to natural log values. Arcsin (angular) transformation was performed on all covariates measured as proportions to “stretch out” proportions close to 0 and 1 [15]. Adherent diversity, congregation diversity, and all remaining variables were analyzed in the meta-regressions in their original metric without any further transformation.

### 2.4. Analyses

Descriptive statistical analyses of data were conducted using Excel (Microsoft Office 365); correlations and collinearity were examined using SPSS v27 (IBM, Armonk, NY, USA). Meta-regression analyses were conducted using Stata v17. The meta-regression analyses were analyzed as random effects models using the Knapp–Hartung variance estimator [16].

The percent of heterogeneity explained by the addition of covariates was estimated as an adjusted R^2^ (expressed as a percent) using Stata’s “meta” suite of commands. The “added value” of religion data was calculated as the difference between the R^2^ of parsimonious meta-regression models with religion variables and the R^2^ of a model without religion variables.

After assessing correlations and collinearity, individual variables were explained as single covariate random effects meta-regression models; multivariate models were then assessed for religion, place (population size and rurality), race and language, age and gender, all demographics, individual behaviors, clinical care, social and economic, physical environment, and all health factors. These full models were then reduced to parsimonious multivariate models. The assessment of added value for religion for demographic and health factors were carried out using parsimonious models.

## 3. Results

### 3.1. Descriptive Statistics

Table 1 reports the descriptive statistics for the county variables in this study as number of counties with data, means, medians, and minimum and maximum values. Also included in this table are the single covariate meta-regression model results reported as percent heterogeneity explained (meta-regression adjusted R^2^ values) and the direction of association and statistical significance (*p* < 0.05) of the covariate reflected by color: yellow—not statistically significant; red—statistically significant in counties with higher mortality; and green—statistically significant in counties with lower mortality).

Counties with higher mortality were statistically significantly (*p* < 0.05) associated with higher values of: congregation density; rurality; African American, American Indian, or Alaskan Native; age 65 years or older; female; smoking; obesity; physical inactivity; sexually transmitted infections; uninsured; preventable hospital stays; unemployment; children in poverty; income inequality; inadequate social support; single parent households; injury deaths; air pollution; drinking water violations; driving alone to work; and long commute when driving alone. Counties with lower mortality were statistically significantly associated (*p* < 0.05) with higher values of: adherent diversity; congregation diversity; population size; Asian, Hispanic, Native Hawaiian, or other Pacific Islander; not-proficient in English; age less than 18 years; food environment index (healthier foods); access to exercise opportunities; excessive drinking; primary care physicians; dentists; diabetes monitoring; mammography screening; high school graduation; some college; and severe housing problems. Variables that were not statistically significant (*p* ≥ 0.05) included: adherents (proportion); White; alcohol-impaired driving deaths; and violent crime.

The single variable explaining the highest between-county mortality heterogeneity was injury deaths (52.5%). Four variables explained between 40 to 50%: children in poverty (49.3%); physical inactivity (49.1%); some college (41.0%); and smoking (40.4%). Three variables explained between 30 to 40%: teen births (39.5%); congregation density (33.2%); and obesity (30.0%). All remaining variables individually explained less than 30%; adherent and congregation diversity explained 11.0% and 9.8%, respectively. 

**Figure 1:**
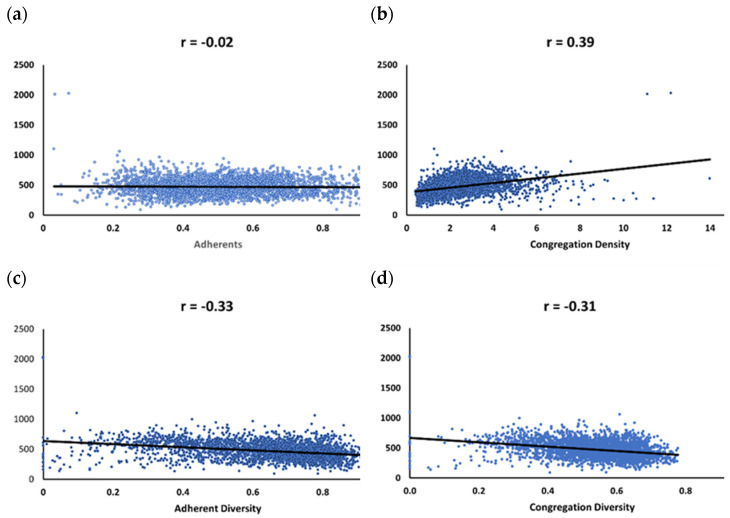
Mortality Correlations and Scatterplots. (**a**) Adherents; (**b**) Congregation Density; (**c**) Adherent Diversity; (**d**) Congregation Diversity.

Figure 1 portrays scatter plots for mortality and the four religion variables, all in the original (not transformed) metrics. Three of the variables (adherents, adherent diversity, congregation diversity) are negatively correlated with mortality, which reflects counties with lower mortality tend to have higher proportions of adherents (*r* = −0.02) and are more religiously diverse both in terms of adherents (*r* = −0.33) and congregations (*r* = −0.31). Counties with higher mortality tend to have greater congregation density (*r* = 0.39) or more congregations per 100 K population.

Table 2 reflects substantial correlations (*r* ≥ 0.60) for the “top ten” (from Table 1) variables explaining heterogeneity as single covariate meta-regression models, and other substantial variable correlations (top half of table), as well as other variables (bottom half). Positive correlation coefficients reflect variables associated with counties having higher mortality rates, and negative with counties have lower mortality rates. Injury deaths, which explains 52.5% of between-county mortality, was not substantially correlated with any other variable. Children in poverty had substantial correlations with four other variables (teen births, college, healthy food, and single parent). Congregations (density) had substantial correlations with three variables (population size, rural, and Asian). Physical inactivity was the third highest in explaining heterogeneity and was substantially correlated with obesity. Hispanic was substantially correlated with non-English proficiency. STI was substantially correlated with Black, White, and single parent. Adherent diversity was substantially correlated with congregation diversity. 

### 3.2. Meta-Regression Results

The variable White (non-Hispanic) had a variance inflation factor (VIF) greater than 10 (as did African American and Hispanic), but when White was removed, all VIF values were less than 10. White was therefore not included in any multivariate model.

Table 3 reflects the progression of individual variables into full and parsimonious multivariate random effects meta-regression models (in this table, the exact value for individual covariates is reported, and color coding, as in Table 1, is again used for all covariates). Variables that were reported by less than 3000 counties were also not included in the multivariate analyses in preference to having multivariate models with at least 3000 counties; these variables were: smoking (*n* = 2711), excessive drinking (*n* = 2225), inadequate social support (*n* = 2471), and violent crime (*n* = 2917).

The transition of specific single covariate variables to parsimonious multivariate models of note (direction of association change) included adherents, congregation diversity, Pacific Islander, age less than 18, sexually transmitted infections, and high school graduation. Specifically, adherents as a single covariate became statistically significant and associated with lower morality in the parsimonious model, while congregation density became statistically not significant. Pacific Islander (Native Hawaiians or other Pacific Islander) changed from being statistically significantly associated with lower mortality to higher mortality; age less than 18 and high school graduation had a similar change. Sexually transmitted infections changed from a statistically significant association with higher mortality to lower mortality. Variables that were statistically significant as single covariates and became statistically significant in the multivariate models included: adherents, and alcohol-impaired driving deaths. The construct than explained that the highest percentage of mortality heterogeneity was social and economic (74.9%), followed by individual behaviors (60.9%), religion (43.2%), race and language (42.6%), age and gender (24.4%), place (19.9%), and the least by physical environment (10.5%).

**Figure 2:**
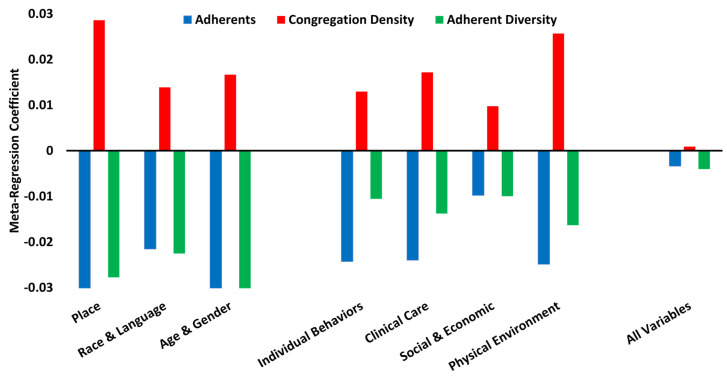
Consistency of Religion–Mortality Direction of Association.

Figure 2 reflects the consistency of the religion–mortality direction of association when controlling for separate demographic and health factor constructs, as well as when controlling for all demographic and health factor constructs simultaneously (using parsimonious models). A higher proportion of adherents and higher values of adherent diversity are consistently associated with lower county mortality (negative regression coefficients). Higher congregation density is consistently associated with higher county mortality (positive regression coefficients).

**Figure 3:**
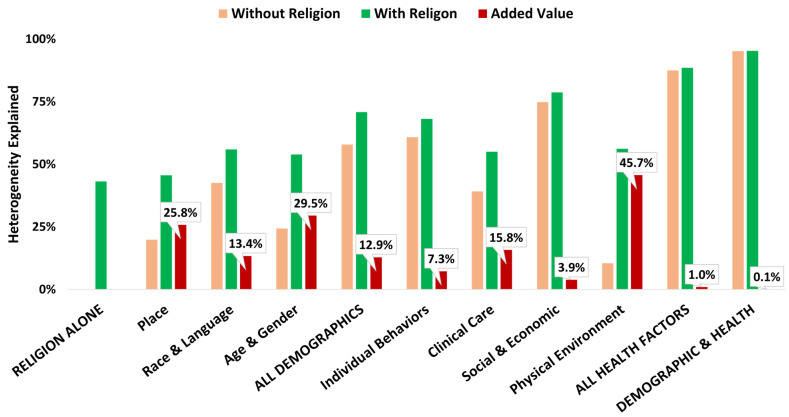
Religion Added Value.

Figure 3 portrays the added value of religion when assessing between-county mortality heterogeneity when controlling for individual demographic and health factor constructs, as well as when controlling for all demographics; all health factors; all demographics and health factors (using parsimonious models). The religion construct alone (Table 3) explains 43.2% of between-county mortality. Depending on how much is explained by separate demographic and health factor constructs, the added value of religion ranges from 7.3% (when added to Social and Economic) to 45.7% (when added to Physical Environment). The added value of religion decreases when added to combined models: 12.9% (when added to all Demographics), 1.0% (when added to all Health Factors), to only 0.1% (when added to all Demographic and Health Factors).

## 4. Discussion

Our research asked questions regarding religion as a population health determinant of mortality variation between U.S. counties. We assessed data from up to 3141 U.S. counties, examining:

1. The direction and strength of any association between religion and mortality, uncontrolled and when controlled for known demographic and health factor mortality determinants.
2. Whether religion explained more, less, or was comparable to demographics and health factors.
3. If the data suggest religion has added value to population health research of mortality.

The best answer to these questions has to be “it’s complicated”. As single covariates or as a single construct, religion is consistently associated with mortality, regardless of what these variables are controlled for. Counties with lower mortality rates are associated with a higher proportion of adherents, greater adherent diversity, and greater congregation diversity. This is consistent with the religion–health evidence at the individual level where religion is a source of social support. Counties with higher mortality rates are associated with higher congregation densities. The only logical explanation for this it that congregations are fulfilling their mission of caring for those who need support. Counties with higher mortality also experience higher rates of poverty, obesity, smoking, teen births, etc.; when life in the present life is dismal at best, it is perhaps natural to hope for something better in the next, and religion may offer that, and thus a greater demand is responded to by greater supply.

However, religion is a label, and much like labels of race, it does not provide an accurate reflection of the complexity of individuals that fall under the label. Therefore, it is not surprising that, upon controlling for health factors that more directly connected with mortality, the strength of association between religion and mortality diminishes. In fact, in this study, once religion was controlled for by demographics and health factors, the “added value” of religion to population health studies of mortality decreases to where religion has an added value of less than one-tenth of a percent. But depending on what data is available to a research project, and if religion data is available, there may still be added value.

There are two important limitations to our research. First, this is an ecological study where counties are the unit of analysis. Readers may be tempted to generalize these findings to individuals, but that is not possible, and we would be remiss in our responsibility if we failed to make note of this. But our interest was in counties, and as an “individual entity”, these data are generalizable to counties or other similar geographical units.

A second limitation is that the adherent and congregation density religion data reflected “any denomination” and thus may not apply to individual denominations. This is supported by the two diversity indices (adherents and congregation) that were both associated with lower mortality. Additional research examining specific denominations is warranted.

In the progression towards parsimonious meta-regression models, several variables dropped out due to statistical non-significance; even when in a single covariate model, they were statistically significant. Highly correlated variables are essentially proxies for each other, and when one drops out in a multivariate model, it does not lose its importance. Smoking and excessive drinking were not included in the movement towards a parsimonious multivariate model because these variables had less than 3000 counties reporting: 2711 and 2225, respectively. As a sensitivity analysis leaving these variables in (and thus reducing the multivariate model number of counties), this parsimonious model (2134 counties) explained 68.5% of between-county mortality, 7.63% points more than the individual behaviors parsimonious model reported in Table 2. In this analysis, smoking was statistically significantly associated with counties with higher mortality, while excessive drinking was significantly associated with counties with lower mortality. Excessive drinking was also significant at the single covariate level, and this association is more likely due to underreporting.

Last, mortality is a single measure of population health. These findings therefore do not generalize to other population health measures. Additional research of religion and other outcomes is also warranted.

## 5. Conclusions

Religion is a complex variable. The measure “adherents” does not assess the depth of one’s faith, nor does it assess the frequency of attending religious services. More importantly, “religion” or “being a religious person” is a label that is not homogeneous. One “religious person” will most likely be substantially different from another “religious person” in terms of factors that are more directly linked to mortality and other health outcomes. The same is true for counties and any other population group. This study was not designed to produce definitive truths but rather to simply ask if religion was worthy of a population health study.

In that regard, this study is a success. The consistency of association between mortality and each of the religion variables, even when controlling for demographic and health factors, is intriguing. The hope of this research was to identify areas worthy of additional study. Why is the relationship consistent? Does the same relationship exist for other population health outcomes? Are the relationships similar between religious denominations? Can population health improvement strategies be more effective when engaging and collaborating with the faith communities?

The study of religion as a determinant of health, either at the individual level or at a population level, has been less extensive in public health than in the clinical sciences; this avoidance recalls the old maxim that cautions any discussion of politics and religion at the dinner table [17]. This avoidance of religion (and politics) in public health circles is ironic given that public health and religion have actually been connected for a long time; this avoidance may be changing. Former Dean of the Emory School of Public Health, James Curran, shares a face of history not well-known regarding John Snow and the removal of the infamous pump handle. Little reference has been given to public health students about Snow’s collaboration with a local parish curate, Reverend Henry Whitehead, whose intimate involvement in the life of the people which led to “understanding how they walked to get water, offering critical insights to how disease spread” [18]. The current Dean of the Boston University School of Public Health, Sandro Galea, reminds us that religion and public health overlap through social justice and the safeguarding of vulnerable populations, and that given the influence of religion and its direct effect on billions of people, public health leaders today recognize how partnerships with religious organizations would help create healthier populations, and public health would be remiss to ignore this opportunity [19].

## Figures and Tables

**Table 1:** County Descriptive Statistics, Single-Covariate Meta-Regression Direction of Association (DA) and Between-County Mortality Heterogeneity Explained (%).

Construct and Variables	Descriptive Statistics					DA ^1^ & %
	*n*	Mean	Median	Low	High	
Health Outcome: Mortality	3141	473.5	463.9	91.2	2028.4	na
Religion						
Adherents	3141	51.3%	49.7%	3.1%	100%	<0.01
Congregation Density		2.4	2.2	0.4	14.0	33.2
Adherent Diversity		0.64	0.69	0	0.96	11.0
Congregation Diversity		0.54	0.56	0	0.78	9.8
Place						
Population Size	3141	98,295.3	25,887	82	9,818,605	14.8
Rurality		58.6%	59.5%	0%	100.0%	19.2
Race and Language						
African American (non-Hispanic)	3141	9.0%	2.2%	0%	85.4%	5.4
American Indian or Alaskan Native		2.1%	0.5%	0%	92.0%	0.3
Asian		1.2%	0.5%	0%	43.8%	29.8
Hispanic		8.3%	3.3%	0.1%	96.4%	8.8
Native Hawaiian or other Pacific Islander		0.1%	0.03%	0%	46.7%	7.0
White (non-Hispanic)		78.7%	86.4%	2.6%	99.0%	<0.01
English language non-proficiency		1.8%	0.8%	0%	29.4%	14.5
Age and Gender						
Age less than 18 years	3141	23.3%	23.2%	0%	40.5%	3.3
Age 65 years and older		15.9%	15.5%	3.2%	35.6%	23.4
Gender female		50.1%	50.5%	26.9%	57.4%	2.0
Individual Behaviors						
Adult Smoking	2711	21.3%	20.8%	3.1%	51.1%	40.4
Adult Obesity	3141	30.6%	30.7%	13.1%	47.9%	30.0
Food Environment Index		7.4	7.6	0	10	13.6
Physical Inactivity		27.8%	28.1%	10.4%	44.9%	49.11
Access to Exercise Opportunities	3117	52.4%	53.6%	0%	100%	16.2
Excessive Drinking	2225	16.5%	16.3%	3.2%	56.2%	13.4
Alcohol-Impaired Driving Deaths	3115	32.0%	31.5%	0%	100%	<0.01
Sexually Transmitted Infections	3135	309.3	231.8	0	2812.9	2.4
Teen Births	3042	41.7	39.9	3.7	123.7	39.5
Clinical Care						
Uninsured	3140	18.5%	18.2%	3.6%	41.4%	11.9
Primary Care Physicians	3017	55.2	50.6	0	458.9	7.8
Dentists	3054	38.4	34.7	0	323.2	14.7
Preventable Hospital Stays	3011	78.8	73.0	19.8	342.4	29.3
Diabetes Monitoring	3094	83.6%	84.7%	16.9%	100%	4.4
Mammography Screening	3055	63.1%	63.6%	30.0%	95%	14.0
Social and Economic						
High School Graduation	3117	82.5%	83.3%	14.0%	100%	5.6
Some College	3141	55.1%	55.1%	2.6%	100%	41.0
Unemployment	3140	9.2%	9.0%	1.6%	29.7%	14.6
Children in Poverty		24.2%	23.5%	2.7%	61.1%	49.3
Income Inequality	3141	4.5	4.4	2.6	9.6	8.7
Inadequate Social Support	2471	19.3%	19.05	0%	100%	15.0
Single Parent Households	3139	31.6%	30.8%	0%	78.7%	21.6
Violent Crime	2917	257.1	202.1	0	1989.5	0.04
Injury Deaths	3020	77.3	73.8	23.7	257.6	52.5
Physical Environment						
Air Pollution—Particulate Matter	3108	11.6	11.9	7.2	14.9	2.6
Drinking Water Violations	3083	9.6%	0.2%	0%	100%	0.7
Severe Housing Problems	3141	14.5%	13.9%	2.2%	71.3%	0.9
Driving Alone to Work		78.2%	79.7%	3.9%	93.9%	7.0
Long Commute—Driving Alone		29.6%	28.7%	0%	71.2%	2.0

^1^ Statistical significance and direction of association (DA) of individual meta-regression coefficients. Red: *p* < 0.05 Higher Mortality; green: *p* < 0.05 Lower Mortality; yellow: *p* ≥ 0.05 Not Statistically Significant.

**Table 2:** Substantial Correlations (*r* ≥ 0.60).

Variable	Mortality R^2^	Variables with *r* ≥ 0.60			
Top 10 Variables: Single Covariate Meta-Regression R^2^s and Other Variables					
Injury Deaths	52.54	none			
Poverty	49.34	0.736 Teen Births	−0.652 College	−0.644 Healthy Food	0.637 Single Parent
Inactivity	49.11	0.713 Obesity			
College	40.97	−0.672 Teen Births			
Smoking	40.44	none			
Teen Births	39.52	0.605 Uninsured			
Congregations	33.24	−0.804 Population	0.695 Rural	−0.606 Asian	
Obesity	30.02	0.713 Inactivity			
Asian	29.75	0.611 Population	−0.601 Rural		
Hospitalization	29.33	none			
Other Variables with *r* ≥ 0.60					
Hispanic		0.847 Language	−0.619 White		
Population		−0.770 Rural			
STI		0.688 Black	−0.675 White	0.661 Single Parent	
Adherent Diversity		0.681 Congregation Diversity			
Black		0.646 Single Parent	−0.635 White		

**Table 3:** Progression from Single Covariate to Parsimonious Multivariate Meta-Regression Models.

Construct and Variables	Single Covariate Model			Multivariate Models					
				Full			Parsimonious		
	*n*	Coeff	%	*n*	Coeff	%	*n*	Coeff	%
Religion									
Adherents	3141	0.001	<0.01	3141	−0.028	43.2	3141	−0.028	43.2
Congregations		0.019	33.2		0.020			0.020	
Adherent Diversity		−0.036	11.0		−0.026			−0.027	
Congregation Diversity		−0.051	9.8		−0.001				
Place									
Population	3141	−0.005	14.8	3141	−0.001	19.9	3141	−0.001	19.9
Rural		0.019	19.2		0.016			0.016	
Race and Language									
Black	3141	0.021	5.4	3141	0.029	42.6	3141	0.029	42.6
Indigenous		0.009	0.3		0.019			0.019	
Asian		−0.162	29.8		−0.164			−0.163	
Hispanic		−0.030	8.8		−0.001				
Pacific Islander		−0.242	7.0		0.102			0.101	
White		0.001	<0.01						
Language		−0.089	14.5		−0.045			−0.049	
Age and Gender									
Age < 18	3141	−0.089	3.3	3141	0.037	24.4	3141	0.037	24.4
Age ≥ 65		0.161	23.4		0.172			0.172	
Female		0.133	2.0		0.056			0.056	
Individual Behaviors									
Smoking	2711	0.160	40.4	3010		60.9	3010		60.9
Obesity	3141	0.221	30.0		0.014				
Healthy Food		−0.006	13.6		−0.002			−0.002	
Inactivity		0.218	49.1		0.147			0.154	
Exercise Access	3117	−0.026	16.2		−0.002			−0.003	
Excess Drink	2225	−0.098	13.4						
Driving Deaths	3115	0.001	<0.01		0.003			0.003	
STI	3135	0.149	2.4		−0.138			−0.132	
Teen Births	3042	0.250	39.5		0.136			0.136	
Clinical Care									
Uninsured	3140	0.090	11.9	2843	0.054	39.8	2925	0.053	39.2
Primary Care	3017	−0.000	7.8		−0.000				
Dentist	3054	−0.000	14.7		−0.000			−0.000	
Hospitalization	3011	0.000	29.3		0.000			0.000	
Diabetes	3094	−0.048	4.4		−0.002				
Mammography	3055	−0.083	14.0		−0.010			−0.012	
Social and Economic									
High School	3117	−0.036	5.6	3009	0.016	74.9	3009	0.016	74.9
College	3141	−0.101	41.0		−0.033			−0.033	
Unemployed	3140	0.138	14.6		0.029			0.029	
Poverty		0.126	49.3		0.046			0.044	
Income	3141	0.008	8.7		−0.000				
Social Support	2471	0.108	15.0						
Single Parent	3139	0.081	21.6		0.028			0.027	
Violent Crime	2917	0.000	0.04						
Injury Deaths	3020	0.001	52.5		0.000			0.000	
Physical Environment									
Air Pollution	3108	0.002	2.6	3056	0.001	10.5	3056	0.001	10.5
Water Quality	3083	0.005	0.7		0.007			0.007	
Poor Housing	3141	−0.025	0.9		−0.012			−0.012	
Drive Alone		0.060	7.0		0.061			0.061	
Long Commute		0.021	2.0		0.016			0.016	

Statistical significance and direction of association of individual and multivariate meta-regression coefficients; variables dropped in multivariate analyses. See Appendix A for variable labels. Red: *p* < 0.05 Higher Mortality; green: *p* < 0.05 Lower Mortality; yellow: *p* ≥ 0.05 Not Statistically Significant; black: variable not included in full and/or parsimonious regression model.

## Data Availability

The data of this study can be provided upon reasonable request to the corresponding author.

## Appendix A

**Table A1:** Data Definitions, Sources, and Data Timeframes (note to self: add more footnotes for special calculations).

**2010 US Religion Census Data ^1^**					
**Variable**	**Label**	**Measure**			
Adherents	Adherents	Percentage of number of persons reported having an affiliation to a congregation (children, members, and attendees who are not members) based on county population size.			
Congregation Density	Congregations	Number of congregations per 100 K based on county population size.			
Population Size	Population	Number of persons living in the county.			
Religion Diversity Index	Adherent Diversity	Diversity of adherents.			Estimated using US Religion Census data.
Denominational Pluralism Index	Congregation Diversity	Diversity of congregations.			
**RWJF Healthy County Data ^2^**					
**Variable**	**Label**	**Measure**	**Data Source & RWJF Report Year(s)**		**Data Timeframes ^3^**
			**Primary Source**	**Year**	
Mortality	Death	Crude death rate per 100 K.	National Center for Health Statistics	2014 & 2015	2008–2010, 2010–2012
**Demographic Data**					
Rurality	Rural	Percentage of population living in a rural area.	US Census Bureau Population Estimates Program (PEP)	2013	2010
African American (non-Hispanic)	Black	Percentage of population that is non-Hispanic African American.	US Census Bureau Population Estimates Program (PEP)	2012, 2013	2009, 2011
American Indian or Alaskan Native	Indigenous	Percentage of population that is American Indian or Alaskan Native.	US Census Bureau Population Estimates Program (PEP)	2012, 2013	2009, 2011
Asian	Asian	Percentage of population that is Asian.	US Census Bureau Population Estimates Program (PEP)	2012, 2013	2009, 2011
Hispanic	Hispanic	Percentage of population that is Hispanic.	US Census Bureau Population Estimates Program (PEP)	2012, 2013	2009, 2011
Native Hawaiian or other Pacific Islander	Pacific Islander	Percentage of population that is Native Hawaiian or other Pacific Islander.	US Census Bureau Population Estimates Program (PEP)	2012, 2013	2009, 2011
White (non-Hispanic)	White	Percentage of population that is non-Hispanic White.	US Census Bureau Population Estimates Program (PEP)	2013	2011
English language proficiency	Language	Percentage of population that is not proficient in English.	American Community Survey (ACS)	2014	2008–2012
Age less than 18 years	Age < 18	Percentage of population below 18 years of age.	US Census Bureau Population Estimates Program (PEP)	2012, 2013	2009, 2011
Age 65 years and older	Age 65+	Percentage of population ages 65 and older.	US Census Bureau Population Estimates Program (PEP)	2012, 2013	2009, 2011
Gender female	Female	Percentage of population that is female.	US Census Bureau Population Estimates Program (PEP)	2012, 2013	2009, 2011
**Individual Behaviors Data**					
Adult Smoking	Smoking	Percentage of adults who are current smokers.	Behavioral Risk Factor Surveillance System (BRFSS)	2014	2006–2012
Adult Obesity	Obesity	Percentage of the adult population (age 20 and older) that reports a body mass index (BMI) greater than or equal to 30 kg/m^2^.	CDC Diabetes Interactive Atlas	2014	2010
Food Environment Index	Healthy Food	Index of factors that contribute to a healthy food environment, from 0 (worst) to 10 (best).	USDA Food Environment Atlas, Map the Meal Gap from Feeding America	2014	2010–2011
Physical Inactivity	Inactivity	Percentage of adults age 20 and over reporting no leisure-time physical activity.	CDC Diabetes Interactive Atlas	2014	2010
Access to Exercise Opportunities	Exercise Access	Percentage of population with adequate access to locations for physical activity.	OneSource Global Business Browser, Delorme map data, ESRI, & US Census Tigerline Files	2014	2010 & 2012
Excessive Drinking	Excess Drink	Percentage of adults reporting binge or heavy drinking.	Behavioral Risk Factor Surveillance System (BRFSS)	2014	2006–2012
Alcohol-Impaired Driving Deaths	Drinking Deaths	Percentage of driving deaths with alcohol involvement.	Fatality Analysis Reporting System	2014	2008–2012
Sexually Transmitted Infections	STI	Number of newly diagnosed chlamydia cases per 100,000 population.	National Center for HIV/AIDS, Viral Hepatitis, STD, and TB Prevention (NCHHSTP)	2013	2010
Teen Births	Teen Births	Number of births per 1000 female population ages 15–19.	National Center for Health Statistics - Natality files	2016	2007–2013
**Clinical Care Data**					
Uninsured	Uninsured	Percentage of population under age 65 without health insurance.	US Census Bureau Small Area Health Insurance Estimates (SAHIE)	2013	2010
Primary Care Physicians	Primary Care	Number of primary care physicians (MD and DO) per 100,000 population.	HRSA Area Resource File	2013	2010
Dentists	Dentists	Number of dentists per 100,000 population.	HRSA Area Resource File	2013	2010
Preventable Hospital Stays	Hospitalization	Rate of hospital stays for ambulatory-care sensitive conditions per 1000 Medicare enrollees.	Dartmouth Atlas of Health Care	2013	2010
Diabetes Monitoring	Diabetes	Percentage of diabetic Medicare enrollees ages 65–75 that receive HbA1c monitoring.	Dartmouth Atlas of Health Care	2013	2010
Mammography Screening	Mammography	Percentage of female Medicare enrollees ages 67–69 that receive mammography screening.	Dartmouth Atlas of Health Care	2013	2010
**Social and Economic Factors Data**					
High School Graduation	High School	Percentage of ninth-grade cohort that graduates in four years.	Individual state Department of Education websites	2012 & 2013	2009–2010 & 2010–2011
Some College	College	Percentage of adults ages 25–44 with some post-secondary education.	American Community Survey, 5-year estimates	2014	2008–2012
Unemployment	Unemployed	Percentage of population ages 16 and older unemployed but seeking work.	Bureau of Labor Statistics: Local Area Unemployment Statistics (LAUS)	2012	2010
Children in Poverty	Poverty	Percentage of people under age 18 in poverty.	US Census Bureau: Small Area Income and Poverty Estimates (SAIPE)	2012	2010
Income Inequality	Income	Ratio of household income at the 80th percentile to income at the 20th percentile.	American Community Survey, 5-year estimates	2015	2009–2013
Inadequate Social Support	Social Support	Percentage of adults without adequate social/emotional support.	Behavioral Risk Factor Surveillance System (BRFSS)	2014	2005–2010
Single Parent Households	Single Parent	Percentage of children that live in a household headed by single parent.	American Community Survey, 5-year estimates	2014	2008–2012
Violent Crime	Violent Crime	Number of reported violent crime offenses per 100,000 population.	Uniform Crime Reporting - FBI	2014	2010–2011
Injury Deaths	Injury Deaths	Number of deaths due to injury per 100,000 population.	CDC WONDER mortality data	2015	2008–2012
**Physical Environment Data**					
Air Pollution—Particulate Matter	Air Quality	Average daily density of fine particulate matter in micrograms per cubic meter (PM2.5).	CDC WONDER Environmental data	2014	2011
Drinking Water Violations	Water Quality	Percentage of population potentially exposed to water exceeding a violation limit during the past year.	EPA Safe Drinking Water Information System (SDWIS)	2013	FY 2012
Severe Housing Problems	Poor Housing	Percentage of households with at least 1 of 4 housing problems: overcrowding, high housing costs, lack of kitchen facilities, or lack of plumbing facilities.	Comprehensive Housing Affordability Strategy (CHAS) data	2016	2008–2012
Driving Alone to Work	Drive Alone	Percentage of the workforce that drives alone to work.	American Community Survey, 5-year estimates	2014	2008–2012
Long Commute—Driving Alone	Long Commute	Among workers who commute in their car alone, the percentage that commute more than 30 min.	American Community Survey, 5-year estimates	2014	2008–2012

^1^ US Religion Census Website: Home | U.S. Religion Census | Religious Statistics & Demographics (usreligioncensus.org); ^2^ Robert Wood Johnson Foundation Healthy County Website: County Health Rankings & Roadmaps; ^3^ When 2 data timeframes are reported (e.g., “2008–2010, 2010–2012” or “2009,2011”, the data from both timeframes are averaged); when a range of years is reported (e.g., 2008–2012, that multiyear timeframe was chosen as the best representing 2010 in the middle of the period; single year timeframes are data from a single year; in some cases, the closest timeframe to 2010 was used for variables that are still current in the RWJF Healthy County model (e.g., FY2012).
